# Epidemiological profile of *Plasmodium ovale* spp. imported from Africa to Anhui Province, China, 2012–2019

**DOI:** 10.1186/s12936-020-03551-8

**Published:** 2021-01-06

**Authors:** Tao Zhang, Shuqi Wang, Duoquan Wang, Sarah Auburn, Shenning Lu, Xian Xu, Jingjing Jiang, Xiaofeng Lyu, Chen Yu, Cuicui Tian, Shizhu Li, Weidong Li

**Affiliations:** 1 Anhui Provincial Center for Disease Control and Prevention, Hefei, 230601 China; 2 National Institute of Parasitic Diseases, Chinese Center for Disease Control and Prevention, Shanghai, 200025 China; 3 Global and Tropical Health Division, Menzies School of Health Research and Charles Darwin University, Darwin, NT Australia

**Keywords:** *Plasmodium ovale* spp., *Plasmodium ovale curtisi*, *Plasmodium ovale wallikeri*, Imported malaria, Anhui province

## Abstract

**Background:**

Although autochthonous malaria cases are no longer reported in Anhui Province, China, imported malaria has become a major health concern. The proportion of reported malaria cases caused by *Plasmodium ovale* spp. increased to levels higher than expected during 2012 to 2019, and showed two peaks, 19.69% in 2015 and 19.35% in 2018.

**Methods:**

A case-based retrospective study was performed using data collected from the China Information System for Disease Control and Prevention (CISDCP) and Information System for Parasitic Disease Control and Prevention (ISPDCP) from 2012 to 2019 to assess the trends and differences between *Plasmodium ovale curtisi* (*P. o. curtisi*) and *Plasmodium ovale wallikeri* (*P. o. wallikeri*)*.* Epidemiological characteristics were analyzed using descriptive statistics.

**Results:**

*Plasmodium o. curtisi* and *P. o. wallikeri* were found to simultaneously circulate in 14 African countries. Among 128 patients infected with *P. ovale* spp., the proportion of co-infection cases was 10.16%. Six cases of co-infection with *P. ovale* spp. and *P. falciparum* were noted*,* each presenting with two clinical attacks (the first attack was due to *P. falciparum* and the second was due to *P. ovale* spp.) at different intervals. Accurate identification of the infecting species was achieved among only 20.00% of cases of *P. ovale* spp. infection. At the reporting units, 32.17% and 6.96% of cases of *P. ovale* spp. infection were misdiagnosed as *P. vivax* and *P. falciparum* infections, respectively.

**Conclusion:**

The present results indicate that the potential of *P. ovale* spp. to co-infect with other *Plasmodium* species has been previously underestimated, as is the incidence of *P. ovale* spp. in countries where malaria is endemic. *P. o. curtisi* may have a long latency period of > 3 years and potentially cause residual foci, thus posing challenges to the elimination of malaria in *P. ovale* spp.-endemic areas. Considering the low rate of species identification, more sensitive point-of-care detection methods need to be developed for *P. ovale* spp. and introduced in non-endemic areas.

## Background

Malaria remains a major global public health concern. In 2018, studies have estimated 228 million malaria cases, leading to 405,000 deaths worldwide [1]. Most malaria cases (93%) and deaths (94%) were in the World Health Organization (WHO) African Region, where *Plasmodium falciparum* is the most prevalent malarial parasite, accounting for approximately 99.7% of cases [1].

In China, the National Malaria Elimination Action Plan (NMEAP) was launched in 2010, with the final objective of achieving elimination by 2020 [2, 3]. Owing to the introduction of the “1-3-7 model” to deliver and monitor the elimination process [4], local transmission of malaria in Anhui, a south-eastern province of China, was successfully interrupted [5]. No autochthonous cases have been reported since 2014. By 2019, Anhui province had been malaria-free for 6 years and passed the sub-national malaria elimination assessment. Nevertheless, with globalization and increased international movement, the province is facing a challenge from imported malaria cases.

In total, 935 imported cases of malaria were reported in Anhui Province from 2012 to 2019. Interestingly, the proportion of malaria caused by *P. ovale* spp. increased during this period to levels higher than expected. *P. ovale* spp. are two of the six *Plasmodium* species causing human transmission and was identified by Stevens in 1922 [6]. Sequence analysis recently revealed that *P. ovale* spp. essentially comprises two nonrecombining species, *Plasmodium ovale curtisi* (*P. o. curtisi*) and *Plasmodium ovale wallikeri* (*P. o. wallikeri*) [7]. Infection due to *P. ovale* spp. has generally been considered rare and limited by geographic distribution [8]. However, the availability of more sensitive PCR diagnosis methods has revealed that its geographic distribution is larger than previously speculated [7, 9]. Thus far, only a few epidemiological studies have investigated the respective dynamics of two sympatric species of *P. ovale* spp. and marked knowledge gaps exist. In the present study, a case-based, retrospective, comparative analysis was performed to analyse the trends and differences between *P. o. curtisi* and *P. o. wallikeri.* An increase in the knowledge of the epidemiology of the *P. ovale* spp. would help develop more effective public health responses.

## Methods

### Cases and sympatric species confirmation

Individuals with a confirmed infection were defined as malaria cases (infections). Since the risk of malaria is high in China, each suspected case is mandatorily reported [10]. Thereafter, staff from the county-level Center collected whole blood samples of patients before antimalarial treatment, and samples were sent to the Malaria Diagnostic Reference Laboratory of Anhui Province. In the reference Laboratory, DNA was extracted using the QIAamp DNA Mini kit (QIAGEN Inc, Hilden, Germany) in accordance with the manufacturer’s instructions. The final diagnosis was confirmed through microscopic examination of Giemsa-stained thick and thin blood films under an oil immersion lens at 1000× magnification, and/or via real-time PCR. Commercial real-time PCR Kits (Shanghai ZJ Bio-tech Co., Ltd, Shanghai, China) were used to distinguish *Plasmodium* species. PCR was performed in a 40-μL reaction mixture \containing 35 μL reaction mix, 0.4  μL enzyme mix, 1 μL internal control, and 4 μL DNA template. The reaction conditions were as follows: 37 ℃ for 2 min and 94 ℃ for 2 min, followed by 40 cycles at 93 ℃ for 15 s and 60 ℃ for 60 s; if *P. ovale* spp. was detected upon species identification, commercial real-time PCR kits (Shanghai BioGerm Medical Biotechnology Co., Ltd, Shanghai, China) were used, designed by referring to a previous study [11], to distinguish between *P. o. curtisi* and *P. o. wallikeri.* Real-time PCR was performed in a 25-μL reaction volume containing a 16-μL reaction mix, 4 μL Primers Probe OVA (c/w), and 5 μL DNA template. The reaction conditions were as follows: 95 ℃ for 5 min, followed by 40 cycles at 95 ℃ for 10 s, and 55 ℃ for 40 s.

### Data collection

Cases of infection acquired outside the country were defined as “imported” [12]. The basic clinical and epidemiological data of each case were obtained from the China Information System for Disease Control and Prevention (CISDCP) and the Information System for Parasitic Disease Control and Prevention (ISPDCP)—a subsystem of the CISDCP, which includes age, sex, occupation, travel history, usual residence, date of onset and diagnosis, symptoms, and prognosis. After the investigation, staff from the county-level Center completed an epidemiological report for disease control primarily to identify the origin of the infection and record other valuable information. In the study, the latency period was determined for each case of malaria, as previously reported, by subtracting the date of arrival in China from the date of symptom onset (the last possible point in time when parasites could have been introduced by the infectious *Anopheles* species. However, this is a minimum estimate [13–15].

### Data analysis

All statistical tests were performed using SPSS version 17.0 (SPSS Inc., Chicago, IL, USA). MapInfo 15.0 (Pitney Bowes Inc., Troy, NY, USA) compiled a thematic map of geographic distribution. The one-sample Kolmogorov–Smirnov test was performed to assess the normality of the distribution of continuous variables. Normally distributed continuous variables are presented as mean ± standard deviation values and non-normal variables were expressed as median and interquartile range (IQR) values. The means of two continuous, normally distributed variables were compared using independent samples Student’s *t*-test. The Mann–Whitney U test was used to compare non-normal variables. Categorical data are presented as ratios and percentages. The differences in proportions were compared using Pearson chi-squared or Fisher’s exact test, as appropriate. All statistical tests were considered significant with 2-sided p value < 0.05.

## Results

### Epidemiological profile of imported malaria in Anhui Province, 2012–2019

**Fig. 1:**
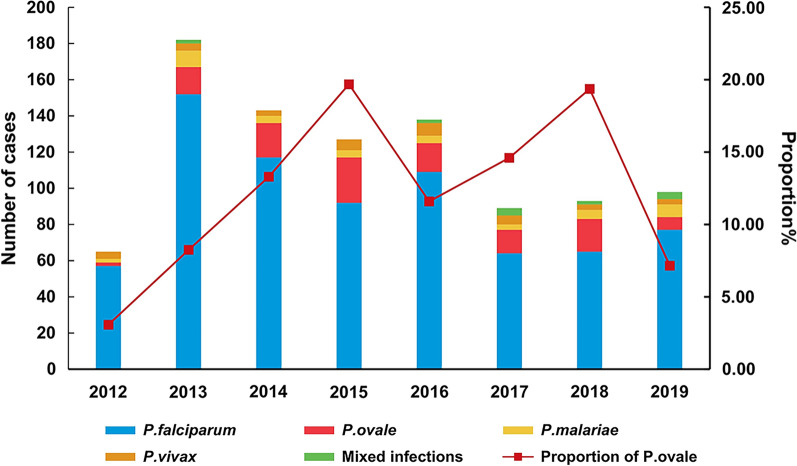
Imported cases of malaria in Anhui Province, 2012–2019

In total, 941 cases of imported malaria were reported in Anhui province from 2012 to 2019. Cross-checks of the patient identifiers revealed that 6 patients had 2 clinical episodes owing to different malarial parasites after returning to China. These patients were reported twice owing to different dates of onset. PCR confirmed that they were co-infections (all were *P. falciparum* co-infected with *P. ovale* spp.). In this study, six cases were recorded as co-infections in accordance with the second clinical episode, and the number of imported malaria cases decreased to 935. *P. falciparum* was the dominant species, accounting for 733 cases (78.4%), followed by *P. ovale* spp. (115 cases, 12.30%), *P. malariae* (38 cases, 4.06%), *P. vivax* (35 cases, 3.74%), and co- infection (14 cases, 1.50%). Among 14 cases of co-infection, there were 12 cases of *P. falciparum* co-infected with *P. ovale* spp., one of *P. falciparum* with *P. malariae,* and one of *P. malariae* with *P. ovale* spp. The proportion of imported cases due to *P. ovale* spp. peaked in 2015 (19.69%) and 2018 (19.35%), and increased from 2012 to 2018 (χ^2^ = 9.626, p = 0.002, excluded 2019) (Fig. 1).

### Origin of imported cases of *P. ovale* spp.

**Fig. 2:**
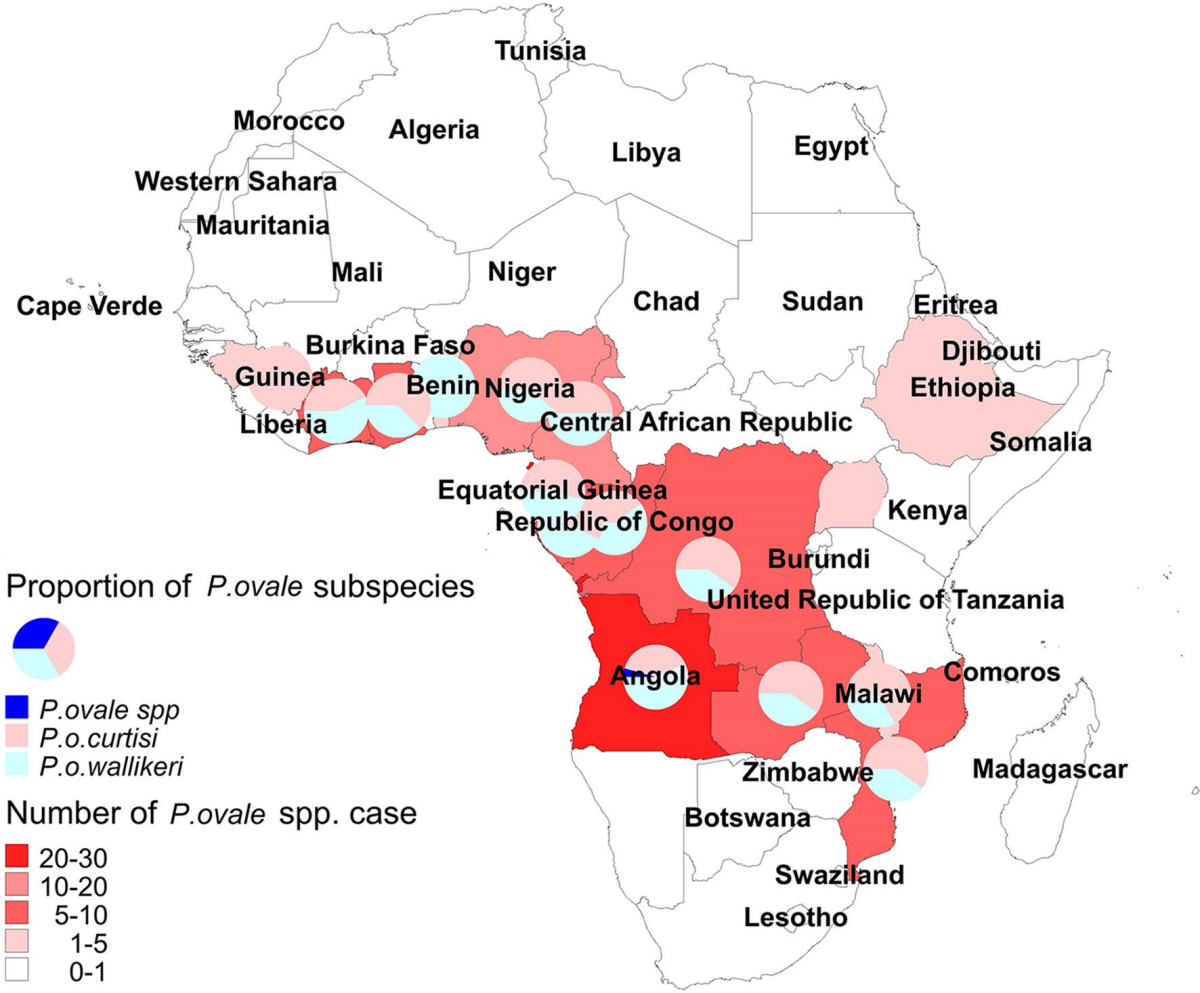
Geographic distribution of the origin of imported cases of *P. ovale* in Africa, 2012–2019

All 128 patients infected with *P. ovale* spp*.,* including co-infections, were imported from 16 countries in Africa. In total, 127 (99.22%) *P. ovale* spp*.* cases were successfully confirmed via PCR; however, an inadequate blood sample was obtained from one patient for PCR and was only confirmed as *P. ovale* spp. through microscopy. The top four countries of origin for these infections were Equatorial Guinea (24, 18.75%), Angola (22, 17.19%), Nigeria (18, 14.06%), and Cameroon (12, 9.38%) (Table 1). One individual, infected in Angola, had a co-infection of *P. o. curtisi* and *P. o. wallikeri*. Therefore, 129 *P. ovale* spp. isolates were included in the analysis. Except for Ethiopia and Uganda (only one case each), *P. o. curtisi* and *P. o. wallikeri* were simultaneously detected in all countries. The proportion of *P. o. curtisi* ranged from 39.13% to 66.67% (Fig. 2).

**Table 1:** Origin of imported *P. ovale* spp. in Anhui, 2012–2019

Country	*P. ovale curtisi*		*P. ovale wallikeri*	
	N	%	N	%
Equatorial Guinea	13	19.12	11	18.33
Nigeria	11	16.18	7	11.67
Angola^a^	9	13.24	13	21.67
Cameroon	6	8.82	6	10.00
Ghana	5	7.35	3	5.00
Gabon	4	5.88	3	5.00
Zambia	3	4.41	2	3.33
Mozambique	3	4.41	2	3.33
Ivory Coast	3	4.41	4	6.67
Congo(Brazzaville)	3	4.41	2	3.33
Malawi	2	2.94	1	1.67
Guinea	2	2.94	0	0.00
Congo(Kinshasa)	2	2.94	3	5.00
Uganda	1	1.47	0	0.00
Ethiopia	1	1.47	0	0.00
Benin	0	0.00	3	5.00
Total	68	100.00	60	100.00

^**a**^One case, infected in Angola, did not have enough blood available to discriminate subspecies of *P. ovale* spp.

### The incidence of *P. ovale* spp. in the primary countries of origin

In general, a high proportion of *P. ovale* spp. did not necessarily indicate a high incidence. In this study, we utilized the number of returnees (Anhui Statistical Yearbook, http://tjj.ah.gov.cn/ssah/qwfbjd/tjnj/index.html) from countries of infectious origin to estimate *P. ovale* spp. *and P. falciparum* incidence rates in the four main countries (Cameroon, Angola, Equatorial Guinea, and Nigeria). Using this method, the estimated average annual incidence rates of *P. ovale* spp. in Cameroon, Angola, Equatorial Guinea, and Nigeria were 2.48%, 0.30%, 1.87%, and 1.70%, respectively. The average annual incidence rates of *P. falciparum* in Cameroon, Angola, Equatorial Guinea, and Nigeria were 9.90%, 2.86%, 8.86%, and 8.11%, respectively (Table 2).

**Table 2:** The incidence rates of *P. ovale* spp. and *P. falciparum* in the population returning from Africa to Anhui province

Year	*P. ovale* spp.				*P. falciparum*			
	Cameroon (%, n/N^a^)	Angola (%, n/N)	Equatorial Guinea(%, n/N)	Nigeria (%, n/N)	Cameroon (%, n/N)	Angola (%, n/N)	Equatorial Guinea(%, n/N)	Nigeria (%, n/N)
2012	0.00 (0/64)	0.00 (0/3648)	0.50 (2/397)	0.00 (0/208)	3.13 (2/64)	0.52 (19/3648)	4.03 (16/397)	2.88 (6/208)
2013	2.3 (1/44)	0.14 (2/1427)	1.53 (5/326)	3.85 (3/78)	25.00 (11/44)	3.22 (46/1427)	10.74 (35/326)	23.08 (18/78)
2014	0.00 (0/60)	0.51 (3/593)	2.65 (7/265)	2.65 (4/176)	11.67 (7/60)	4.72 (28/593)	12.08 (32/265)	7.39 (13/176)
2015	2.15 (2/93)	1.02 (5/489)	5.38 (6/112)	0.00 (0/205)	1.08 (1/93)	7.16 (35/489)	10.71 (12/112)	5.37 (11/205)
2016	4.73 (4/85)	1.21 (3/249)	0.00 (0/74)	3.41 (5/147)	12.94 (11/85)	16.47 (41/249)	5.41 (4/74)	7.48 (11/147)
2017	4.76 (2/42)	1.00 (1/101)	3.88 (2/52)	5.00 (5/100)	11.90 (5/42)	12.87 (13/101)	11.54 (6/52)	10.00 (10/100)
2018	5.71 (1/18)	3.69 (6/163)	18.18 (1/6)	0.00 (0/87)	16.67 (3/18)	5.52 (9/163)	66.67 (4/6)	13.79 (12/87)
Average annual incidence (%)	2.48	0.30	1.87	1.70	9.90	2.86	8.86	8.11

^a^ n was the number of reported imported cases, and N was the number of people returning from the infectious origin countries in the same year

The incidence rate is underestimated because the number of people returning from Africa comes from official statistics, in which it is impossible to include all overseas laborers. Therefore, the results are for reference only

### Epidemiological characteristics of *P. o. curtisi and P. o. wallikeri*

**Fig. 3:**
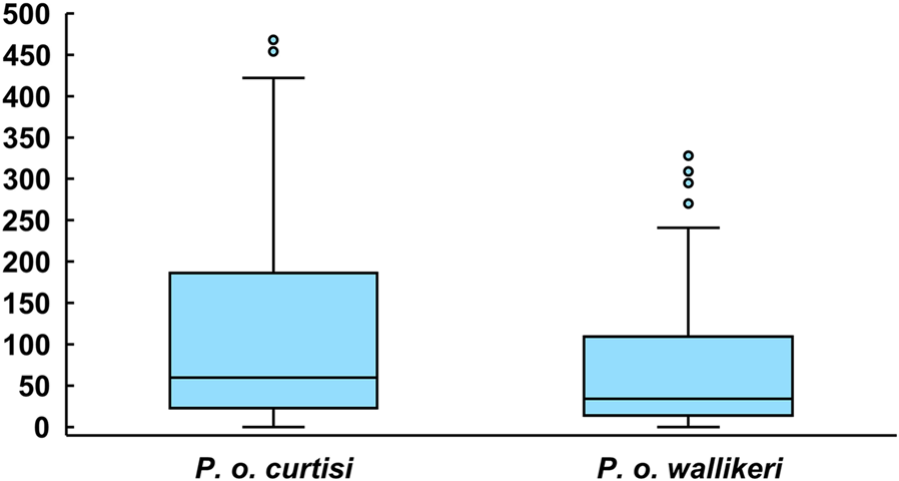
Latency period for cases of *P. ovale curtisi* and *P. ovale wallikeri* in Anhui province, 2012–2019

Of 128 patients infected with *P. ovale* spp.*,* 113 were single-species infections, as determined through PCR. Sixty-two (62/113, 54.87%) cases of *P. o. curtisi* and the remaining 51 cases of *P. o. wallikeri* (51/113, 45.13%) were noted. Among the other 15 cases, there were 13 cases of co-infections, 1 case with co-infection of *P. o. curtisi* and *P. o. wallikeri,* and 1 case without species confirmation. The median latency period for *P. o. curtisi* (59.50 d, IQR: 23.0–192.75) was slightly but not significantly longer than that of *P. o. wallikeri* (34 d, IQR: 12–112.50) (*P* = 0.070) (Fig. 3, Table 3). In the study, the longest latency period of *P. ovale* spp. was 1299 days, which was noted in an individual co-infected with *P. o. curtisi* and *P. falciparum*. Furthermore, no significant differences in sex, age, occupation, or history of malaria were observed between the *P. o. curtisi* and *P. o. wallikeri* groups (Table 3). In this study, co-infections of *P. ovale* spp. and other species, predominantly *P. falciparum* (12 cases) were reported in 13 cases in total. One case presented a co-infection of *P. ovale* spp. and *P. malariae.* The co-infection rate was 10.16% (13/128). Of all 12 co-infection of *P. ovale* spp. and *P. falciparum* cases, six had only one clinical episode and six had two clinical attacks, whereby the first attack was due to *P. falciparum* and the second attack was due to *P. ovale* spp.. The intervals between the 2 clinical episodes were 33, 56, 127, 204, 295, and 1279 d, for the six patients, respectively. Among patients with one clinical episode, 4 were infected with *P. o. wallikeri* and two were infected with *P. o. curtisi*; among patients with two clinical episodes, 3 cases were of *P. o. wallikeri* and 3 were *P. o. curtisi*.

**Table 3:** Epidemiologic characteristics of imported two *P. ovale* subspecies

Variables	*P. ovale curtisi *(n = 62) N (%)	*P. ovale wallikeri *(n = 51) N (%)	P value
Sex^a^			
Male	61 (98.4)	51 (100.0)	1.000
Female	1 (1.6)	0 (0.0)	
Age(years), mean(SD)	43.84 ± 9.21	43.53 ± 7.82	0.850
Occupation^a^			
Worker	51 (82.3)	46 (90.2)	0.463
Waiter	7 (11.3)	3 (5.9)	
Other	4 (6.5)	2 (3.9)	
Previous malaria			
Yes	44 (71.0)	41 (80.4)	0.248
No	18 (29.0)	10 (19.6)	
Duration of stay overseas^a^^,^^b^			
≤ 30	0 (0.0)	1 (2.3)	0.489
≤ 180	6 (11.8)	7 (16.3)	
≤ 365	10 (19.6)	11 (25.6)	
> 365	35 (68.6)	24 (55.8)	
Latency period (days)^c^, median (IQR)	59.50(23.0–192.75)	34 (12–112.50)	0.070

^a^Differences in proportions were tested by Fisher’s exact test

^b^94 cases (83.19%; 94/113) with available information of duration of stay overseas

^c^The time elapsed, in days, between arrival in China and onset of disease was defined as the latency period. Cases showing onset of symptoms before arrival were not included in the analysis. A case with a record of 1533 days is excluded, as the authenticity is questionable

### Diagnosis and clinical characterization of imported *P. ovale* spp. infections

To analyze the diagnostic data on imported *P. ovale* spp. infections, individuals with a *P. falciparum* infection constituted the control group in the study. The median latency period of *P. ovale* spp. was 49 (IQR: 16.5–169.5) d and that of *P. falciparum* was 6 (IQR: 2–10) d; The median interval from onset to the first medical visit for *P. ovale* spp. was 1 (IQR: 0–3) d and that for *P. falciparum* was 1 (IQR: 0–2) d. The median interval from onset to the first medical visit to the diagnosis of *P. ovale* spp. infections was 1 (IQR: 0–3) d, while that for *P. falciparum* was 1 (IQR: 0–2) d. Based on the differences in the median intervals between the two species, *P. ovale* spp. had a significantly longer latency than *P. falciparum* (Z = −12.947, p < 0.001).

The parasite identification rates for *P. ovale* spp. and *P. falciparum* were comparable at 93.04% and 93.59%, respectively. However, the rates of species identification were significantly different between *P. ovale* spp. and *P. falciparum* (χ^2^ = 255.841, p < 0.001). Furthermore, among the 115 *P. ovale* spp. cases, only 20% of the cases (23/115) had an accurate species identification on microscopy (Table 4), while 32.17% (37/115) of cases were misdiagnosed as *P. vivax*, 6.96% (8/115) as *P. falciparum*. In the remaining 40.87% (47/115) of cases, species identification was not attempted, and only the parasite was identified (positive or negative finding). Among 733 *P. falciparum* cases, species identification revealed accurate results in 86.90% of cases (637/733); however, results were not obtained for 11.87% (87/733) of cases. Only 9 cases were misdiagnosed with other malarial parasite species or with co-infections.

**Table 4:** The diagnosis of imported *P. ovale* and *P. falciparum* in Anhui, 2012–2019

Variables	*P. ovale* (n = 115) N (%)	*P. falciparum* (n = 733) N (%)	P value
Latency (days), Median (IQR)^a^	49 (16.5, 169.5)	6 (2, 10)	< 0.001
Time from onset to first medical visit (days), Median (IQR)	1 (0, 3)	1 (0, 2)	0.479
Time from first medical visit to diagnosis (days), Median (IQR)	1 (0, 3)	1 (0, 2)	0.091
Parasite detection^b,c^	107 (93.04)	686 (93.59)	0.826
Species identification^c,d^	23 (20.00)	636 (86.77)	< 0.001

^a^Cases with onset of symptoms before arrival were not included in the analysis

^b^The results of diagnosis came from the reporting units

^c^Parasite detection: calculated according to whether or not a parasite was detected [13]

^d^Species identification: calculated based on the number of correctly identified species [16]

Of the 115 individuals with *P. ovale* spp. infection, common clinical symptoms included fever (99.13%), chills (82.61%), sweating (59.13%), and headaches (55.65%). Furthermore, 53.91% cases presented with typical clinical manifestations of malaria (chills, fever, and sweating).

## Discussion

Although *P. ovale* spp. was identified by Stevens in 1922 [6], it has received limited attention in medical research. In 2010, two sympatric species*, P. ovale curtisi* and *P. ovale wallikeri*, were confirmed through sequence analysis [7]. However, few studies have described their epidemiological differences and have not reported consistent results. A recent study of imported cases in the United Kingdom reported that *P. o. wallikeri* had a shorter latency than *P. o. curtisi* [13], concurrent with another study on imported cases in Henan Province, China [14]. However, another study of imported cases in Jiangsu Province, China, did not report a significant difference in the latency period [15]. In this study, the difference in the latency period of two sympatric species was slightly short of statistical significance (p = 0.070), suggesting that *P. ovale wallikeri* has a shorter latency periods; however, larger studies are needed to confirm these results at the statistical significance threshold. In the study, the longest latency period of *P. ovale* spp. was 1299 d, which was noted in a case of a co-infection with *P. o. curtisi* and *P. falciparum*. These data are concurrent with the longest latency periods reported by Nolder et al. (1083 days) [13] and Zhou et al. (1265 days) [14]. The long latency periods of *P. o. curtisi* are worthy of attention, probably resulting in residual foci. It would be challenging to eliminate malaria in *P. ovale* spp.-endemic areas. Moreover, the concept of a latency period in *P. ovale* spp. has been challenged owing to limited experimental and clinical data supporting the hypnozoite model*.* Richter et al. reported that evidence was not sufficient to unequivocally demonstrate that *P. ovale* spp. hypnozoites are found in the human host [17]. Another review reported only 18 cases of relapse for *P. ovale* spp. in nearly 100 years [18]. However, a recent study provides direct evidence, using molecular analyses, of the reemergence of *P. ovale curtisi* strains, concurrent with the currently accepted relapse theory. Interestingly, relapse of *P. ovale wallikeri* infections was not noted in this study [19]. In this study, co-infections of *P. ovale* spp. */ P. falciparum* were noted, wherein two clinical attacks were triggered (the first attack due to *P. falciparum,* and the second due to *P. ovale* spp.). At the primary *P. falciparum* attack, patients were administered artemisinin-based drugs without primaquine, the only effective drug against dormant liver parasites [20]. The second clinical attack due to *P. ovale* spp. occurred at a different interval. The remaining *P. ovale* spp. infections were treated with primaquine after diagnosis and no parasites reemerged in the blood samples (after the final diagnostic finding of the Malaria Diagnostic Reference Laboratory of Anhui Province feedback, *P. ovale* spp. were treated with primaquine to prevent relapse). Our findings potentially support the existence of hypnozoites of *P. ovale* spp. Moreover, the six cases of co-infections with two clinical episodes constitute the most important findings of this study. Identification of such co-infections is difficult in an endemic transmission setting. As *P. ovale* spp. infections are no endemic in China, if the present patient does not travel abroad again, it should be easy to diagnose co-infections. Furthermore, long-term observation would be required. In contrast, in endemic areas, especially in the high transmission setting, numerous cases of malaria were reported. In this context, if the interval between two clinical attacks was too long, it would be difficult to link these clinical events together, probably being considered independent. These six cases strongly suggest that the potential of *P. ovale* spp. to co-infect with other malarial species has been underestimated because cases as such were difficult to come across in cross-sectional surveys. Data from two recent studies provide evidence regarding this perspective [21, 22]. It is worth noting that 71.0% *P. ovale curtisi* and 80.4% *P. ovale wallikeri* are associated with a previous history of malaria, suggesting that more co-infections, presenting with two clinical attacks, may not be reported. Because our surveillance system only recorded the second clinical attack due to *P. ovale* spp. within the country, and not abroad, the proportion of *P. ovale* spp. may have increased. Nonetheless, more evidence is required. Mehlotra et al.reported that the higher overall prevalence of *P. falciparum* and *P. vivax* in a human population is associated with fewer mixed infections than expected [23], using point-prevalence data from 35 other studies. Based on our results, a potential explanation is that a moderate proportion of co-infections involving hypnozoites was undetected.

McKenzie and Bossert et al. reported that infection by one *Plasmodium* species does not reduce the susceptibility to infection by other species [24]. Therefore, an increase in the rate of co-infections suggests that the prevalence of *P. ovale* spp. has been underestimated. In this study, we examined the returnees from four African countries to estimate the incidence rates of *P. ovale* spp. and *P. falciparum*. Although it is only an estimate, with *P. falciparum* as a reference, the data suggest that the incidence of *P. ovale* spp. was higher than expected and a growing body of evidence supports this hypothesis [25–27]. Considering the geographic distribution, our findings suggest that the two sympatric species, *P. o. curtisi* and *P. o. wallikeri,* have been circulating simultaneously in numerous Africa countries, concurrent with a previous study [28].

Owing to their similarities in morphology and life cycle, *P. ovale* spp. is easily and frequently misdiagnosed as a *P. vivax* infection [29, 30]. In this study, only 20.00% of *P. ovale* spp. cases (23/115) returned an accurate species identification on microscopic examination; 32.17% (37/115) of cases were misdiagnosed as *P. vivax* infections and 6.96% (8/115) of cases were misdiagnosed as *P. falciparum* infections. For the remaining 40.87% (47/115) of cases, species identification was not attempted, and only the results of parasite identification (positive or negative findings) were obtained. The success rates of species identification were significantly different between *P. ovale* spp. (20.00%) and *P. falciparum* (86.77%), Probably because two rapid diagnostic tests (Pf/pan, by Wondfo and by ACCESSBIO) highly sensitive to *P. falciparum*, but insensitive to *P. ovale* spp. [31, 32], have been used to provide parasite-based diagnosis in Anhui province since 2013. Misdiagnosis of *P. ovale* spp. infections as *P. vivax* or *P. falciparum* infections may have led to inappropriate case management measures and treatment regimens. Therefore, more sensitive point-of-care detection methods for *P. ovale* spp. need to be developed and introduced in non-endemic areas.

Our study has several limitations. First, it was a case-based, retrospective study, and was subject to recall bias. Second, some differences were simply beyond the threshold for statistical significance, requiring larger studies to confirm them. Lastly, the data on the use of prophylaxis are limited, thus potentially affecting the latency period of *P. ovale* spp.

## Conclusion

In this study, 6 cases of co-infections of *P. ovale* spp.*/P. falciparum* were noted, which presented as two clinical attacks, indicating that the potential of *P. ovale* spp. to co-infect with other malarial species has been previously underestimated. The incidence of *P. ovale* spp. has also probably been underestimated in these source countries where the disease is endemic. *P. o. curtisi* may have a long latency period of > 3 years and potentially cause residual foci, thus posing challenges to the elimination of malaria in *P. ovale* spp.-endemic areas.

Given the low rate of species identification, more sensitive point-of-care detection methods need to be developed for *P. ovale* spp. and introduced in non-endemic areas.

## Data Availability

The datasets used and/or analysed during the current study are available from the corresponding author on reasonable request.
